# Virulence Genes of *Helicobacter pylori* Increase the Risk of Premalignant Gastric Lesions in a Colombian Population

**DOI:** 10.1155/2022/7058945

**Published:** 2022-09-28

**Authors:** Yeison Carlosama-Rosero, Claudia Acosta-Astaiza, Carlos H. Sierra-Torres, H. Bolaños-Bravo, Andrés Quiroga-Quiroga, Juan Bonilla-Chaves

**Affiliations:** ^1^Interdisciplinary Research Group on Health and Disease, Cooperative University of Colombia, Pasto, Colombia; ^2^Human and Applied Genetics Research Group, University of Cauca, Popayán, Colombia

## Abstract

**Background:**

Genetic variability of *Helicobacter pylori* is associated with various gastrointestinal diseases; however, little is known about interaction with sociodemographic in the development of premalignant lesions in Colombian patients.

**Methods:**

An analytical study was conducted including cases (patients with gastric atrophy, intestinal metaplasia, and gastric dysplasia) and controls (patients with nonatrophic gastritis). Sociodemographic information was obtained using a questionnaire. Histopathological diagnosis was performed according to the Sydney System. The cagA and vacA genotypes were established using polymerase chain reaction in paraffin blocks. The effect of each variable on the study outcome (premalignant lesion) is presented as odds ratio (OR) and 95% CI. A *p* value of <0.05 was considered as statistically significant.

**Results:**

The vacA/s1m1 genotype increases the risk of developing premalignant lesions of the stomach (OR: 3.05, 95% IC: 1.57–5.91, *p*=0.001). Age and educational level showed a positive interaction with the s1m1 genotype (adjusted OR: 3.68, 95% CI: 1.73–7.82, *p*=0.001). The cagA genotype was not correlated to the development of premalignant lesions of the stomach (OR: 1.32, 95% CI: 0.90–1.94, *p*=0.151).

**Conclusions:**

The vacA genotype, age, and educational level are indicators of the risk of developing premalignant lesions of the stomach in the study population. *Significance Statement*. Genetic variability of *H. pylori* and sociodemographic information could be used to predict the risk of premalignant lesions in stomach in Colombian population.

## 1. Introduction

Gastric cancer (GC) is a high-impact disease at the global level. According to GLOBOCAN, in 2020, 1,089,103 cases of gastric cancer were reported worldwide, making it the fifth most prevalent cancer and the third leading cause of death due to cancer [1] Colombia is one of the countries with the highest incidence of GC, along with Japan, Costa Rica, Singapore, Korea, and Chile [2]. As reported by the National Administrative Department of Statistics, the Cauca Department has one of the country's highest standardized mortality rate: 10.7/100,000 inhabitants/year for female and 18.7/100,000 inhabitants/year for male.

Besides its high incidence, late diagnosis of gastric cancer is one of the critical factors for mortality in Cauca. Adrada et al. showed that the proportion of cancer diagnosed at advanced stages in Cauca was >90% [3]. Unfortunately, in most cases, its occurrence results in mortality due to the disease, with 5-year survival rates of <10% [4]. Currently, prevention and early detection are the optimal strategies for mitigating the effects of the disease.

Considering these strategies, the carcinogenesis theory proposed by Dr. Correa is particularly significant because it addresses the onset of histopathological lesions that precede the development of gastric cancer [5, 6]. According to this theory, intestinal adenocarcinoma—the most common histotype in developing countries—is preceded by a series of progressive histopathological changes that begin with chronic atrophic gastritis, intestinal metaplasia, and gastric dysplasia [7]. However, only a small proportion of patients with these lesions eventually develop cancer, with a higher risk associated with gastric dysplasia (6%) and lower risk associated with gastric atrophy (0.1%) [8].

It is challenging to predict the risk of progression, and this risk can be modulated by various genetic and environmental factors including genetic variability of *Helicobacter pylori* (*H. pylori*) [9, 10]. For example, the *vacA*/s1m1 and *cagA*+ genotypes have been shown to be associated with an increased risk of presenting precursor lesions of gastric malignancy [11–16]. It should be noted that the role of *Helicobacter pylori* in the development of peptic ulcer, stomach cancer, and in some forms of gastric lymphoma has been well documented over the past decades, which has been summarized and discussed in a recent very comprehensive review [17].

In Colombia, bacterial genotypes have been associated with the onset of premalignant lesions of the stomach in few studies [18–20]. In these studies, the approach is based on comparing genotype frequencies, and the results showed contradictory findings. However, to the best of the authors' knowledge to date, no recent research has evaluated the interactive effect of the genetic variability of *H. pylori* and sociodemographic factors. Therefore, the aim of the present study was to estimate the association between *H. pylori* genotypes and sociodemographic factors with the development of precursor lesions of gastric malignancy (atrophy, metaplasia, and dysplasia) in a population from Cauca, Colombia.

## 2. Materials and Methods

An unpaired case-control analytical study was conducted with patients admitted to the Gastroenterology Units of the San José University Hospital and Endovideo in the city of Popayán from January 2008 to December 2014. Samples were collected by convenience sampling, and included patients were aged >18 years with a histopathological diagnosis of nonatrophic gastritis (controls) and those with precursor lesions of gastric malignancy (cases). Inclusion criteria included the following: participants should be born in a municipality of Cauca; should be children of parents from Cauca; and should be diagnosed with *H. pylori* infection determined by histopathological tests and corroborated by molecular diagnostics [polymerase chain reaction (PCR)]. Participants with a history of gastric surgery, who received treatment for *H. pylori* infection, who had an HIV infection, and who had gastric adenocarcinomas other than of the intestinal histotype were excluded. Prior to sample collection, participants voluntarily signed an informed consent form and were interviewed via a survey to obtain information on sociodemographic and clinical variables.

### 2.1. Histopathological Analysis and Molecular Evaluation of Bacterial Genotypes

Gastric samples were collected by experienced gastroenterologists via gastrointestinal endoscopy. Patients underwent gastrointestinal endoscopy following referral for dyspeptic symptoms and after fasting for at least 8 h. Although participants were not sedated, they received topical oropharyngeal anesthesia. Five samples corresponding to two antral biopsies (major and minor curvature), two corpus biopsies (major and minor curvature), and one incisura angularis biopsy were obtained. These samples were fixed in buffered formalin and stained with hematoxylin-eosin and Giemsa stains. Two pathologists conducted histopathological analyses of the samples. Patients were assigned to four diagnostic categories: nonatrophic gastritis, atrophic gastritis, intestinal metaplasia, and gastric dysplasia. The most severe lesion was selected as the final diagnosis in each patient. The visual analog scale was used according to the Sydney System. Genotyping studies of *H. pylori* were performed using DNA extraction of paraffin-embedded biopsies using the Chelex® 100 technique (No. C7901; Sigma, St. Louis, MO). PCR previously described by Yamaoka et al. [18] was performed for the amplification of vacA genes. PCR mixtures were prepared using 50 ng of genomic DNA, 100 *μ*mol of dNTPs, 2.5 *μ*L of 10× PCR buffer, 1.0 mM MgCl_2_, 1 U of Taq DNA polymerase (No. M1665; Promega, Madison, WI), and 30 pmol of each primer shown in Table 1.

The reactions commenced with a denaturation at 95°C for 1 min, followed by 35 cycles of denaturation at 94°C for 1 min, hybridization at 52°C for 1 min, extension at 72°C for 1 min, and final extension at 72°C for 10 min. The obtained products were analyzed using electrophoresis in 2% agarose gels at 80 V for 40 min, and the genotypes were identified according to the expected base pair size, additionally, those samples with more than one allele (s/m) were considered as coinfection and were grouped under this same category for the analyses.

The *H. pylori* strains NCTC-11637 and NCTC-11638 as well as the clinical isolate 3062 were provided by the Colombian National Cancer Institute and used as positive controls. The PCR tests included adequate internal amplification controls and molecular markers (ladders).

### 2.2. Bias Control

To control biases associated with sociodemographic information, biologists and doctors who belonged to the Human and Applied Genetics Research Group were trained for standardizing questions in a closed questionnaire, which was completed before gastrointestinal endoscopy.

To reduce biases associated with histopathological information, diagnoses were validated by a different pathologist who was unaware of the previous diagnosis. In cases wherein diagnosis differed, the case was jointly re-evaluated to reach diagnostic consensus. To limit disagreement in cases of gastric dysplasia, they were grouped into a single category that included low- and high-grade gastric dysplasia.

On the other hand, the histopathological diagnosis of *H. pylori* infection was corroborated using Giemsa staining and PCR. *H. pylori* infection was concluded by minimum one positive result PCR for Vac-A and the presence of bacteria in histopathological examination. Molecular diagnostics were conducted according to globally accepted protocols; the equipment was calibrated, and pilot tests were performed to verify the quality of reagents and extraction kits.

To determine the behavior of variables for each histopathological lesion, the frequencies of each precursor lesion of gastric malignancy were compared with the control group. Thereafter, patients with precursor lesions were regrouped as a single category for comparison with the control group.

### 2.3. Statistical Analysis

Mean differences in age were evaluated using one-way ANOVA along with post hoc tests. Differences in proportions were evaluated using the chi-squared test of independence. Odds ratio (OR) and *p*-values were used to evaluate the effect of each variable of interest on the response variable (precursor lesion of gastric malignancy). Multivariate logistic regression analysis was performed including the variables that met the Hosmer–Lemeshow criteria and those reported in the scientific literature as significant variables. A *p*-value of <0.05 and 95% CI were considered statistically significant. Data were analyzed using SPSS® version 23.

## 3. Results

Of a total of 821 patients, 452 met the inclusion criteria. Intestinal metaplasia was the most prevalent precursor lesion of malignancy. Patient distribution according to histopathological diagnosis were nonatrophic gastritis 188 (41.59%), atrophy 62 (13.71%), metaplasia 164 (36.28%), and dysplasia 38 (8.42%).

The average patient age was 43 years for the nonatrophic gastritis group, 53 years for the atrophic gastritis, 54 years for the metaplasia group, and 62 years for the dysplasia group. When average age was compared using ANOVA single-factor test, significant differences were observed among all groups (*p*=0.001). Post hoc analysis showed significant differences between each precursor lesion of gastric malignancy with chronic nonatrophic gastritis (*p*=0.001).

Regarding age, the most prevalent age group in the control category was 18–45 years; in the atrophic gastritis and metaplasia group, the prevalent age group was 46–60 years, whereas in the dysplasia group, the prevalent age group was >60 years. Patient distribution according to age, sex, origin, income, education level, and ethnicity is shown in Table 2.

Each precursor lesion of gastric malignancy was compared with the chronic nonatrophic gastritis group to estimate the measures of association. Female sex, age of 18–45 years, urban origin, ≥1 CLMMW income, college/higher education, and mestizo ethnicity groups were selected as control categories. Analyses showed significant associations between the age groups of >45 and >60 years and elementary school level in all the precursor lesion of gastric malignancy, also the male sex and rural origin has significant association in metaplasia and dysplasia lesions, the <1 CLMMW income only has significant association in dysplasia (Table 3).

To study the *vacA* genotypes, the s1, s2, m1, and m2 alleles were examined and grouped according to their virulence profile. Analysis showed the presence of the s2m2 genotype in 43 patients, which included 28 (65.12% isolates) with nonatrophic gastritis, 8 (18.6% isolates) with gastric atrophy, 6 (13.95% isolates) with intestinal metaplasia, and 1 (2.33% isolate) with gastric dysplasia. On the other hand, the s1m1 genotype was the most prevalent in the study population, being present in 358 patients, which included 136 with chronic nonatrophic gastritis, 43 with gastric atrophy, 145 with intestinal metaplasia, and 34 with gastric dysplasia. Cases of coinfection wherein the presence of a *cagA*+ bacterium was documented were considered the *cagA*+ category. *cagA* and *vacA* genotype distribution according to the type of diagnosis is shown in Table 4.

To facilitate analyses, patients with gastric atrophy, intestinal metaplasia, and gastric dysplasia were grouped into a single category (precursor lesions of gastric malignancy) and compared with the control group. Chi-squared test showed significant differences among the *vacA* genotypes (*p*=0.02) and showed no differences regarding the *cagA* genotype (*p* > 0.05). To analyze the measures of association, the *vacA*/s2m2 and *cagA*- genotypes were selected as control categories (Table 5).

Patients with gastric atrophy, intestinal metaplasia, and gastric dysplasia were grouped into a single category (precursor lesions of gastric malignancy) and compared with the control group to obtain the crude OR of the sociodemographic variables. Only significant variables (categorized age, sex, origin, income category, and education level) were included in this analysis according to the findings of bivariate analysis by diagnostic categories (Table 3). In a previous analysis, *cagA*+ gene and <1 CLMMW income showed no significant associations either with the crude estimate (*cagA*+ OR: 1.32, 95% CI: 0.903–1.94, *p*=0.151; <1 CLMMW income OR: 1.42, 95% CI: 0.919–2.19, *p*=0.142) or in the model adjusted for genotypes and age (*cagA*+ adjusted OR: 1.19, 95% CI: 0.74–1.86, *p*=0.486; <1 CLMMW income adjusted OR: 0.884, 95% CI: 0.507–1.54, *p*=0.667. Moreover, the variable male sex and rural origin showed no significant associations in the adjusted OR (male sex adjusted OR: 1.19, 95% CI: 0.74–1.89, *p*=0.471; rural origin adjusted OR: 1.09, 95% CI: 0.66–1.82, *p*=0.721). Considering these data, to evaluate the most parsimonious model, *cagA* gene, income, sex, and origin category variables were excluded from the final logistic regression model.  Table 6 illustrates the risk of developing precursor lesions of gastric malignancy according to age-adjusted *vacA* genotypes and education level.

## 4. Discussion

An association between age and onset of premalignant lesions of the stomach was determined in this study. The findings showed that the prevalence of gastric dysplasia is higher in patients older than >60 years of age, whereas injuries, such as gastric atrophy and intestinal metaplasia are more prevalent in patients aged 46–60 years of age. Similar results have been reported by other authors [21, 22] showing a direct correlation between the severity of precursor lesions of gastric malignancy and age.

The greatest age-related risk is due to genomic instability acquired over the years due to chronic inflammation, cumulative damage by free radicals, and inefficiency of DNA repair mechanisms [23–26]. On the other hand, normal gastric mucosa reportedly lacks telomerase activity, and a progressive increase in the activity of this enzyme is directly related to premalignant lesions and cancer [27]. Other studies as well as the present investigation suggest that preneoplastic lesions represent histological changes caused by tissue aging and dysfunctional adaptive responses, thereby increasing the risk of tumors.

The present study showed a significant association between education level and the presence of preneoplastic lesions. Elementary school (OR: 3.36, 95% CI: 1.83–6.16) and middle school (OR: 2.26, 95% CI: 1.18–4.31), increase the risk of developing premalignant lesions. These findings are not consistent with those reported by Flores-Luna et al. for Mexico, Paraguay and Colombia [28]. In contrast, Mouw and cols reported that patients with a lower education level had nearly a 70% increased risk of GC as compared with patients with the highest education level [29]. The StoP study assessed the risk between GC and educational level and reported a higher risk of developing intestinal GC in individuals with the lowest level of education [30]. It is possible that improving education contributes to individuals being more aware of their health and probably, helps reduce the risk of gastric cancer [31].

Regarding bacterial genotypes, the s1m1 genotype was more prevalent in the case group, whereas the s2m2 genotype was more prevalent in the control group. Similar results have been reported by Colombian and foreign authors [32–34]. The role of the s1m1 genotype can be explained via different mechanisms such as the synthesis of a vacuolizing protein, which induces greater epithelial damage, development of a more persistent inflammation, and blockage in the proliferation of T lymphocytes via its arrest in the G1 or S phase of the cell cycle [35, 36].

In a recent meta-analysis, 33 studies were evaluated, which overall included 2697 controls and 1446 cases with gastric cancer and precursor lesions of gastric malignancy. In that study, the s1 allele showed an increased risk of gastric atrophy (RR: 1.11 95% CI: 1.019–1.222) and intestinal metaplasia (RR: 1.41, 95% CI: 1.03–1.94). Furthermore, the m1 vacA allele was associated with intestinal metaplasia (RR: 1.57, 95% CI: 1.24–1.98); however, there was no documented increase in the risk of gastric atrophy [37]. The same study showed that adjusting the model for the incidence standardized by age decreased the association of bacterial genotypes with gastric cancer. Although *p*-values revealed significant associations in data analysis, the lower limit of 95% CI of the s1 allele was extremely close to the null hypothesis value. In contrast, the results of the present investigation showed significant associations of the s1m1 genotype with 95% CI far from the null hypothesis both in bivariate analysis (OR: 3.05, 95% CI: 1.57–5.91) and age-adjusted multivariate logistic regression model (adjusted OR: 3.94, 95% CI: 1.88–8.23).

Similarly, the analysis of genotype distribution by diagnostic category (Table 4) helps conclude that the prevalence of the s1m1 subtype increases with the rise in the severity of premalignant lesions, whereas the opposite seems to occur for the s2m2 genotype, suggesting a proportional relationship between the severity of the lesion and bacterial genotype. These findings highlight the conceptual value of the carcinogenesis model proposed by Dr. Correa and provide an important theoretical basis for its predictive capacity for cancer risk.

The carcinogenic effect of the *cagA* gene product is attributable to diverse mechanisms such as the reorganization of the cytoskeleton of epithelial cells, change in cell phenotype, and activation of signaling pathways that stimulate cell proliferation [38–40]. These mechanisms would partly explain a higher incidence of gastric cancer in populations wherein approximately 90% isolates are *cagA*+ and a lower incidence of gastric cancer wherein the prevalence of *cagA*+ is lower [41, 42]. In the present study, the prevalence of the *cagA*+ genotype between case and control groups did not significantly differ, and a relationship between the *cagA* genotype and development of precursor lesions of gastric malignancy was not documented. These results differ from those reported in the literature [16, 43, 44]. A possible explanation for this finding is related to polymorphisms of the *cagA* gene, phosphorylated EPIYA motifs and *cagA* multimerization motifs (CM). It has been proposed that polymorphisms of the *cagA* gene and phosphorylated EPIYA motifs modulate the risk of diseases, such as duodenal ulcer, degree of inflammation, and risk of GC [45, 46]. A study conducted in Nariño revealed that Colombian strains with two and three EPIYA-C motifs and western CM motif were associated with more severe gastric lesions [47]. On the other hand, dietary factors can modulate the risk of gastric carcinogenesis by modifying host mucosal factors, regulating inflammatory response, or inducing epigenetic changes [48]. For example, the effect of salt intake on *H. pylori* virulence has been evaluated in microbiological, transcriptional, and proteomic studies, showing changes in bacterial morphology and a higher transcription of the *cagA* gene under high-salt concentration conditions [49–51]. A higher carcinogenic effect related to salt intake and *cagA* overexpression has been demonstrated in animal models [52]. Interestingly, epidemiological studies, as well as many in vivo and in vitro studies have suggested that *Allium* vegetables and their constituents can be effective in the prevention and inhibition of the progression of carcinogenesis due to the numerous possible beneficial properties of the above-mentioned substances [53]. At the light of these findings, EPIYA-C, CM motifs, salt intake and vegetable consumption should be evaluated in the future to better determinate disease risk in our population.

In a study, we previously reported the association between *vacA* s1m1 genotype (OR: 6.18; CI: 1.25–30.51; *p*=0.025) in patients older than 50 years with pathological diagnosis of gastric cancer (dysplasia and gastric cancer) compared with patients with chronic nonatrophic gastritis [54]. Unlike this, the present research explores the risk of *H. pylori* genotypes and sociodemographic factors from early preneoplastic lesions (atrophy, intestinal metaplasia). Our results support that the s1m1 genotype is associated with the precursor lesions of gastric malignancy and this association is strengthened with an increase in age and lower education level. In addition, it can be concluded that the severity of premalignant lesions of gastric malignancy is directly correlated with advanced age and cytotoxic *H. pylori* genotypes.

The limitations of this study could be selection bias, since it is not a population-based study and the controls were obtained from the same consultation of symptomatic patients, so prevalence could not be determined. In addition, the development of cancer is a multifactorial disease and there may be other host or environmental factors that may influence the development of this pathology; therefore, further studies that may include other variables such as host polymorphisms, environmental exposure, are recommended. An earlier version of this manuscript has been presented as preprint [55].

## Figures and Tables

**Table 1:** Primer sequence for polymerase chain reaction amplification.

Genes and regions		Sequence (5′-3′)	Size (bp)
*vacA* s1/s2	VAI-F	ATGGAAATACAACAAACACAC	259 (s1)/286 (s2)
	VAI-R	CTGCTTGAATGCGCCAAAC	

*vacA* m1/m2	VAG-F	CAATCTGTCCAATCAAGCGAG	570 (m1)/645 (m2)
	VAG-R	GCGTCAAAATAATTCCAAGG	

*cagA*	cagA-F	GATAACAGGCAAGCTTTTGAGG	349
	cagA-R	CTGCAAAAGATTGTTTGGCAGA	

**Table 2:** Distribution of participants in the study groups.

		CNAG^†^	Atrophy	Metaplasia	Dysplasia	Total
Age	18–45 years	119 (63.3)	13 (21)	40 (24.4)	4 (10.5)	176 (38.9)
	46–60 years	58 (30.8)	30 (48.4)	78 (47.6)	13 (34.2)	179 (39.6)
	≥61 years	11 (5.9)	19 (30.6)	46 (28)	21 (55.3)	97 (21.5)

Sex	Male	56 (29.8)	20 (32.3)	68 (41.5)	23 (60.5)	167 (36.9)
	Female	132 (70.2)	42 (67.7)	96 (58.5)	15 (39.5)	285 (63.1)

Origin	Urban	132 (70.2)	41 (66.1)	82 (50)	16 (42.1)	271 (60)
	Rural	56 (29.8)	21 (33.9)	82 (50)	22 (57.9)	181 (40)

Income	<1 CLMMW^‡^	136 (72.3)	45 (72.6)	129 (78.7)	34 (89.5)	344 (76.1)
	≥1 CLMMW	52 (27.7)	17 (27.4)	35 (21.3)	4 (10.5)	108 (23.9)

Education level	Elementary school	68 (36.2)	37 (59.7)	112 (68.3)	30 (78.9)	247 (54.6)
	Middle school	56 (29.8)	17 (27.4)	38 (23.2)	6 (15.8)	117 (25.9)
	College/higher education	64 (34)	8 (12.9)	14 (8.5)	2 (5.3)	88 (19.5)

Ethnicity	Black	2 (1.1)	0	2 (1.2)	0	4 (0.9)
	Indigenous	8 (4.3)	3 (4.8)	4 (2.4)	1 (2.6)	16 (3.6)
	Caucasian	1 (0.5)	0	1 (0.6)	0	2 (0.4)
	Mestizo	177 (94.1)	59 (95.2)	157 (95.7)	37 (97.4)	430 (95.1)
	Total	188 (100)	62 (100)	164 (100)	38 (100)	452 (100)

^†^CNAG: chronic nonatrophic gastritis; ^‡^CLMMW: current legal minimum monthly legal salary in force wage.

**Table 3:** Odds ratio (OR) of sociodemographic factors.

		Atrophy		*p*	Metaplasia		*p*	Dysplasia		*p*
		OR	IC 95%		OR	IC 95%		OR	IC 95%	
Age	18–45 years	1	1	1	1	1	1	1	1	1
	46–60 years	4.73	(2.3–9.75)	0.001	4	(2.44–6.55)	0.001	6.67	(2.08–21.35)	0.001
	≥61 years	15.81	(6.19–40.38)	0.001	12.4	(5.88–26.3)	0.001	56.79	(16.52–195.25)	0.001

Sex	Female	1	1	1	1	1	1	1	1	1
	Male	1.12	(0.61–2.08)	0.714	1.67	(1.07–2.59)	0.023	3.61	(1.76–7.44)	0.001

Origin	Urban	1	1	1	1	1	1	1	1	1
	Rural	1.21	(0.65–2.23)	0.546	2.36	(1.52–3.65)	0.001	3.24	(1.58–6.63)	0.001

Income	≥1 CLMMW^‡^	1	1	1	1	1	1	1	1	1
	<1 CLMMW	1.01	(0.532–1.92)	0.971	1.41	(0.86–2.3)	0.171	3.25	(1.1–9.61)	0.033

Education level	College/Higher education	1	1	1	1	1	1	1	1	1
	Elementary school	4.35	(1.88–10.05)	0.001	7.53	(3.92–14.45)	0.001	14.12	(3.24–61.49)	0.001
	Middle school	2.43	(0.97–6.06)	0.057	3.1	(1.52–6.31)	0.002	3.43	(0.66–17.67)	0.141

Ethnicity	Mestizo	1	1	1	1	1	1	1	1	1
	Indigenous	1.67	(0.203–13.78)	0.633	1.88	(0.19–18.77)	0.59	0.94	(0.102–8.68)	0.958
	Caucasian	NA^§^	NA	NA	NA	NA	NA	NA	NA	NA
	Black	NA	NA	NA	NA	NA	NA	NA	NA	NA

^‡^CLMMW: current legal minimum monthly wage. ^§^NA = not applicable. A *p* value of <0.05 was considered statistically significant.

**Table 4:** Bacterial genotype distribution in the study groups.

		CNAG^†^	Atrophy	Metaplasia	Dysplasia	Total
*vacA* genotypes	s1m1	136 (72.3)	43 (69.4)	145 (88.4)	34 (89.5)	358 (79.2)
	s1m2	2 (1.1)	3 (4.8)	4 (2.4)	0	9 (2)
	s2m2	28 (14.9)	8 (12.9)	6 (3.7)	1 (2.6)	43 (9.5)
	Coinfection^‡^	22 (11.7)	8 (12.9)	9 (5.5)	3 (7.9)	42 (9.3)

*cagA* genotypes	*cagA*−	116 (61.7)	39 (62.9)	82 (50)	24 (63.2)	261 (57.7)
	*cagA*+	72 (38.3)	23 (37.1)	82 (50)	14 (36.8)	191 (42.3)
	Total	188 (100)	62 (100)	164 (100)	38 (100)	452 (100)

^†^CNAG: chronic nonatrophic gastritis. ^‡^Coinfection: s1s2m1, s1s2m2, s1m1m2, s2m1m2, s1s2m1m2.

**Table 5:** Measures of association of *vacA* and *cagA* bacterial genotypes.

		Nonatrophic gastritis	Precursors lesions of gastric malignancy	OR	IC 95%	*p* value
vacA	s2m2	28 (14.9)	15 (5.7)	1	1	1
	s1m1	136 (72.3)	222 (84)	3.05	(1.57–5.91)	0.001
	s1m2	2 (1.1)	7 (2.7)	6.53	(1.2–35.48)	0.03
	Coinfection^‡^	22 (11.7)	20 (7.6)	1.69	(0.71–4.06)	0.234

cagA	*cagA*−	116 (61.7)	145 (54.9)	1	1	1
	*cagA*+	72 (38.3)	119 (45.1)	1.32	(0.903–1.94)	0.151

Patients with atrophy, intestinal metaplasia, dysplasia, were added to one category. ^‡^Coinfection: s1s2m1, s1s2m2, s1m1m2, s2m1m2, s1s2m1m2. A *p* value of <0.05 was considered statistically significant.

**Table 6:** Multivariate logistic regression model showing measures of association between the variables of interest and outcome (precursor lesions of gastric malignancy).

Variable	Crude		*p*-value	Adjusted^a^		*p*-value	Adjusted^b^		*p*-value
	OR	95%CI		OR	95%CI		OR	95%CI	
Age									
46–60 years	4.35	(2.79–6.79)	0.001	3.85	(2.38–6.23)	0.001	4.91	(3.09–7.78)	0.001
≥61 years	16.32	(8.08–32.95)	0.001	11.85	(5.65–24.85)	0.001	17.09	(8.35–34.99)	0.001

Education level									
Elementary school	7.02	(4.07–12.12)	0.001	3.36	(1.83–6.16)	0.001	NA^§^	NA	NA
Middle school	2.9	(1.6–5.26)	0.001	2.26	(1.18–4.31)	0.013	NA	NA	NA

Genotypes									
VacA/s1m1	3.05	(1.57–5.91)	0.001	3.68	(1.73–7.82)	0.001	3.94	(1.88–8.23)	0.001
VacA/s1m2	6.53	(1.2–35.48)	0.03	5.86	(0.905–38.01)	0.064	7.53	(1.11–50.88)	0.038
Coinfection^‡^	1.69	(0.71–4.06)	0.234	1.98	(0.73–5.35)	0.178	1.97	(0.74–5.19)	0.173

a: adjusted for categorized age, bacterial genotype, and education level; b: adjusted for age and bacterial genotype. ^§^NA: not applicable. ^‡^Coinfection: s1s2m1, s1s2m2, s1m1m2, s2m1m2, s1s2m1m2. A *p*-value of <0.05 was considered statistically significant.

## Data Availability

The date used to support the findings of this study are included within the article.
